# A Study of the Relationship Between Nurses’ Professional Self-Concept and Professional Ethics in Hospitals Affiliated to Jahrom University of Medical Sciences, Iran

**DOI:** 10.5539/gjhs.v8n4p82

**Published:** 2015-07-30

**Authors:** Nehleh Parandavar, Afifeh Rahmanian, Zohreh Badiyepeymaie Jahromi

**Affiliations:** 1 Research center for social Determinants of Health, Jahrom University of Medical Sciences, Jahrom, Iran; 2 Department of Nursing, Jahrom University of Medical Sciences, Jahrom, Iran

**Keywords:** Self-Concept, nurses, professional ethics, nursing profession

## Abstract

**Background:**

Commitment to ethics usually results in nurses’ better professional performance and advancement. Professional self-concept of nurses refers to their information and beliefs about their roles, values, and behaviors. The objective of this study is to analyze the relationship between nurses’ professional self-concept and professional ethics in hospitals affiliated to Jahrom University of Medical Sciences.

**Methods:**

This cross sectional-analytical study was conducted in 2014. The 270 participants were practicing nurses and head-nurses at the teaching hospitals of Peimanieh and Motahari in Jahrom University of Medical Science. Sampling was based on sencus method. Data was collected using Cowin's Nurses’ self-concept questionnaire (NSCQ) and the researcher-made questionnaire of professional ethics.

**Results:**

The average of the sample's professional self-concept score was 6.48±0.03 out of 8. The average of the sample's commitment to professional ethics score was 4.08±0.08 out of 5. Based on Pearson's correlation test, there is a significant relationship between professional ethics and professional self-concept (P=0.01, r=0.16).

**Conclusion:**

In view of the correlation between professional self-concept and professional ethics, it is recommended that nurses’ self-concept, which can boost their commitment to ethics, be given more consideration.

## 1. Introduction

As an independent field of study in medical sciences, nursing is committed to providing optimal medical, clinical, and therapeutic services to maintain and improve health in the society (Jolaee et al., 2010).

Ethics have become integral to satisfactory nursing and nurses are expected to observe the standard professional ethics and principles that are intrinsic to a medical relationship (Tefag, Nikbakht Nasrabadi, Mehran, & Dinmohammadi, 2004). All nurses, regardless of their specialties, need guidance in making ethical decisions at work: if ethics are ignored, nurses’ professional performance will suffer (Hassanpoor, Hosseini, Fallahi Khoshknab, & Abbaszadeh, 2011). Nursing graduates sometimes doubt if the care they provide is in accordance with professional ethics, which results in low self-confidence and stress (Borhani, Alhani, Mohammadi, & Abbaszadeh, 2011), and may even cause them to request transfer. On the other hand, acting in patients’ interests and observing ethics result in positive psychological reactions, such as fulfillment, higher motivation, and feelings of competence (Hassanpoor et al., 2011). Accordingly, there is a list of ethical dos and don’ts which are intended to ensure that nurses observe values and norms, and serves as a model for and criterion of behavior toward patients (Lashkar Bloki, 2008). Nurses should actively behave based on their professional ethics, so that they can act efficiently during clinical decision-making. Thus, ethics play a major part in clinical care (Tefagh et al., 2004; Brunner, Smeltzer, & Bare, 2009).

Professional self-concept is the conception that an individual has of his/her profession (Shafi Abadi, 2007); in other words, it is an individual's information and beliefs about his/her role, values, and behavior (Takase, Kershaw, & Burt, 2002). Professional self-concept serves as a framework for determining individuals’ roles in their social positions. How professional and valuable nurses feel depends on their understanding of themselves and of their performance as nurses (Fagermoen, 1997; Secrest, Norwood, & Keatley, 2003). Our images of ourselves influence reality, and a positive self-image is the prerequisite of a positive self-concept (Porter & Porter, 1991). Nurses’ attempts to adapt to various situations and people and behave in the best way depend on their images of themselves (Leddy & Pepper, 1993). Self-concept is essential to feeling able and the ability to show that in one's professional performance; poor self-concept results in individuals’ underestimating themselves and failure to use their knowledge, skills, and professional capabilities (Adib HajBagheri, Salsali, & Ahmadi, 2006). Accordingly, it is believed that a high self-concept contributes to nurses’ ability to perform their tasks (Hensel & Stoelting-Gettelfinger, 2011).

It should be noted that nursing education in Iran has undergone significant change since its genesis with foreign missionaries over one hundred years ago. More recently, following the 1979 Islamic revolution, nurse education has followed the direction taken by most other countries in moving from an apprenticeship model of training to an academic model. A series of transformative changes to nursing education specifically-and across the higher education system generally-has resulted in nurses now being able to undertake study across all university-based programs up to and including doctoral level. Contemporary nursing students have access to full-text professional journals through the internet, and they may pursue their doctoral studies in other countries. Although these improvements in nursing education in Iran are to be applauded, much more needs to be accomplished to ensure that highly competent nurse practitioners continue to be produced in this country (Khomeiran & Deans, 2007).

### 1.1 Research Aim

The objective of this study is to determine nurses’ professional self-concept and professional ethics in hospitals affiliated to Jahrom University of Medical Sciences and then analyze the relationship between them.

## 2. Method

### 2.1 Participants

This cross sectional-analytical study was conducted in 2014. The sample was composed of nurses and head-nurses practicing in hospitals affiliated to Jahrom University of Medical Sciences. Sampling was based on census method. Information was collected from nurses who were willing to participate and had at least 6 months’ experience in clinical care.

### 2.2 Procedure

The co-researcher, after explaining the objectives of the study to the authorities at the two hospitals, visited all the wards including internal, surgical, emergency, children's, and intensive care units to gather data. The questionnaires were distributed in various working shifts. The questionnaires, handed out all at once, were completed by the nurses at their leisure and returned.

### 2.3 Questionnaire

Data was collected using a three-part questionnaire: personal information, nurse self-concept questionnaire (NSCQ) and professional ethics questionnaire. The nurse self-concept questionnaire consists of 36 items which address the following six aspects: nurse general self-concept (6 items), knowledge (6 items), care (6 items), leadership (6 items), Staff relations (6 items) and communication (6 items). Each item is an affirmative statement, and scored from 1 to 8 based on the 8-point Likert scale, with higher points indicating higher levels of self-concept. In Hensel and Stoelting-Gettelfinger's study (2010), the Cronbach's alpha of the questionnaire was reported to be between 0.87-0.91, and in Cowin, Johnson, Craven and Marsh's study (2008), the correlation coefficient of the questionnaire was reported to be high and its Cronbach's alpha was 0.82-0.95. The validity and reliability of the questionnaire have been verified in Iran (Badiyepeymaye Jahromi, Keshavarzi, & Jahanbin, 2013).

The professional ethics questionnaire was designed by the researcher: after a review of the available books and articles on medical ethics and ethical codes, the researcher extracted information about the aspects in question and designed 38 items in 7 areas: respecting patients’ rights (14 items), patient education (4 items), respecting the other members of the medical team (5 items), responsibility (6 items), conflict management (3 items), commitment to confidentiality (3 items), and justice in health care (3 items). The face validity and content validity of the questionnaire—the relevance and comprehensibility of the questions—were verified by 10 professors at Jahrom Nursing Department, and its Chronbach's alpha was calculated to be 0.95. The items on the questionnaire were scored from 1 (very low) to 5 (very high), with higher scores indicating higher commitment to professional ethics.

### 2.4 Data Analysis

Data were entered into SPSS version 16.0. To analyze the distribution of the data, the researcher used descriptive statistics, such as frequencies, means, and standard deviations, and to analyze the relationships among the variables of the study, Pearson correlation coefficient was used. It must be noted that the incomplete questionnaires were left out.

### 2.5 Ethical Considerations

Regarding ethical considerations, the plan of the study was approved by the Research Committee and Committee of Ethics at Jahrom University of Medical Sciences. Introduction papers issued by the Research Department were produced at the hospitals where the study was conducted. Also, in accordance with research ethics, the researcher explained the objectives of the study to the sample under study and assured them that their information would remain confidential, their names were not required, and the participants were asked to give their informed consent.

## 3. Results

Out of the 270 distributed questionnaires in nurses who qualified to participate, 261 were fully completed and used for analysis. 203(77.8%) of the studied nurses were female. The averages of the nurses’ ages and experiences were 33.77±0.57 and 8.35±0.44, respectively. Their other demographic characteristics are shown in Table 1.

**Table 1:** Nurses’ demographic characteristics

Demographic characteristics		N (%)
**Sex**	Female	203 (77.8%)
	Male	58 (22.2%)
**Type of employment**	formal	33 (12.6%)
	Contract	206 (79%)
	Corporate	12 (4.6%)
	Planned nurse	10 (3.8%)
**Education**	Bs	234 (89.6%)
	Ms	27 (10.4%)
**Position**	Head-nurse	30 (11.5%)
	Nurse	231 (88.5%)

Bs= Bachelor degree; Ms= Master of Science.

The average of the sample's professional self-concept score was 6.48±0.03 out of 8. The highest professional self-concept score—6.70±0.06—was related to the aspect of nurses’ General Self Concept, and the lowest score—6.06±0.04—was related to leadership. The average of the sample's commitment to professional ethics score was 4.08±0.08 out of 5 (Table 2).

**Table 2:** The mean and standard deviation of nurse self-concept, professional ethics and its dimensions

Nurse Self-Concept Questionnaire	Mean ± SD
**Total professional self-concept**	6.48±0.03

Nurse General Self Concept	6.70±0.06
Care	6.57±0.03
Knowledge	6.59±0.07
Staff Relations	6.66±0.05
Communications	6.32±0.03
Leadership	6.06±0.04

**Professional Ethics Questionnaire**	**Mean ± SD**

**Total professional ethics**	4.08±0.08

respecting patients’ rights	3.88±0.03
patient education	4.02±0.06
respecting the other members of the medical team	4.83±0.48
responsibility	3.92±0.02
conflict management	3.88±0.03
commitment to confidentiality	3.90±0.03
justice in health care	4.03±0.03

Based on Pearson's correlation test, there is a significant relationship between professional ethics and professional self-concept (P=0.01, r=0.16). There was not a statistically significant relationship between any of the areas of professional self-concept and the average of the total score of commitment to professional ethics. There was only a slightly significant relationship in the area of relationship with one's co-workers (P<0.001, r: 0.24) (Table 3).

**Table 3:** Correlation of total professional ethics and nurse self-concept dimensions

Nurse Self-concept dimensions	Total professional ethics	

	r	p-value
Nurse General Self Concept	0.10	0.09
Care	0.05	0.37
Knowledge	0.10	0.11
Staff Relations	0.24	P<0.001*
Communications	0.08**-**	0.90
Leadership	0.03	0.63

In reverse analysis, also, there was no significant relationship between total professional self-concept and any of the aspects of professional ethics, except respect for the other members of the medical team (P<0.01, r: 0.15) (Table 4).

**Table 4:** Relationship between total professional self-concept and professional ethics dimensions

Professional ethics dimensions	Total Nurse self-concept	

	r	p-value
respecting patients’ rights	0.12	0.06
patient education	0.04	0.51
respecting the other members of the medical team	0.15	0.01*
responsibility	0.09	0.14
conflict management	0.02	0.64
commitment to confidentiality	0.12	0.05
justice in health care	0.22	0.07

## 4. Discussion

A professional nurse is expected to be qualified for correct and safe practice (Borhani et al., 2011). The results of the present study,—which was an attempt to examine the relationship between nurses’ professional self-concept and commitment to professional ethical in the hospitals affiliated to Jahrom University of Medical Sciences,—show that in view of the maximum score of the self-concept questionnaire, the nurses under study achieved high professional self-concept scores: 6.48 out of 8. In his study, Hensel (2008) reported the average of the professional self-concept scores of 84% of the nurses with over 10 years’ experience as high.

It has been shown that graduate nurses with positive professional self-concept are very likely to stay in their first jobs for at least a year (Siebens et al., 2006; Cowin & Hengstberger-Sims, 2006). Though job satisfaction is cited as an important factor in staying in a job, the study of Cowin et al. (2008) have shown that professional self-concept is a more reliable indicator. Concerning professional ethics, also, the nurses in the present study obtained high scores: 4.08 out of 5. The existence of a relationship between these two variables can show that professional self-concept contributes to individuals’ commitment to ethical codes.

“Responsibility” is one of the areas of commitment to professional ethics questionnaire. In their study entitled “A Study of Nurses’ Commitment to Professional Ethics in Case of Medicines Administration,” Tefagh et al. (2004) discovered that most of the sample performed poorly while administering medicines. Since administering medicines is part of a nurse's responsibilities, the poor scores in the aforementioned area are in agreement with the results of the study of Tefagh et al.

Hassanpour et al. (2011) studied the influence of teaching nursing ethical codes on nurses’ ethical sensitivity during decision-making in the public hospitals of Kerman, Iran; they discovered a significant change in nurses’ ethical sensitivity during decision-making before and after intervention. It appears that educational interventions in the area of medical ethics, even regardless of professional self-concept, can positively affect individuals’ performances in the case of their commitment to professional ethics. Study results show that professional self-concept can further the influence of such interventions. In a study of the effects of teaching professional self-concept on senior nursing students’ clinical performance, there was a significant improvement in the students’ clinical performance immediately, and three months after intervention, which is an indication of the positive impact of positive professional self-concept on nurses’ clinical performance (Jahanbin, Badiyepeyma, Sharif, Ghodsbin & Keshavarzi, 2012). Thus, using educational interventions to call attention to the importance of professional self-concept and ethics will prove effective and, regarding the correlation between professional self-concept and professional ethics, enhancing one will improve the other one.

The study of Leuter, Petrucci, Mattei, Tabassi and Lancia (2013) confirm the above conclusions: in their study, they interviewed 374 nurses to explore the ethical problems in nursing, the educational lacks, and nurses’ attitudes to application of ethical sources; the nurses reported that they were faced with many sensitive ethical issues and lacked proper ethical support and education programs on ethics. These factors can affect individuals’ commitment to ethical codes in any profession and need to be studied more deeply.

In their study of the perception of Sudanese and Chinese nurses of ethical issues, Silen, Tang and Ahlström (2009) studied 136 Chinese and 137 Sudanese nurses. Despite the similarities in their statements and the common belief that current nursing is far from satisfactory, the nurses from the two countries had significantly different opinions about ethical issues: the Sudanese nurses were more concerned about ethics. It should be noted that such factors as religion, nationality, and professional environment can significantly influence professional self-concept and commitment to professional ethics. In a study, Peter, Macfarlane and O’Brien-Pallas (2004) analyzed the dominant ethical characteristics of Canadian nurses’ professional environments; the results showed that the stressful environment lead to major ethical problems and the nurses perceived their professional environment as ethically unstable. A mutual understanding of this issue can affect the professional self-concept of practicing nurses and even nursing students. So, it is important that professional environments be made less stressful.

Arthur and Randle (2007) and Cowin et al. (2008) believe that development of a healthy professional self-concept will result in nurses’ more efficient care and a less stressful environment for nurses. Likewise, Hensel et al. (2011) has shown that changing the level of stress in clinical environments leads to a change in the level of professional self-concept, which is due to the fact that stress can potentially have adverse effects on professional self-concept. Less stressful professional environments can contribute to professional self-concept. Relationships among co-workers can bring about such conditions: the results of the present study show that there are significant relationships between the self-concept aspect of “relationship with one's co-workers” and professional ethics, and the ethical aspect of “respect for the other members of the medical team” and professional self-concept. Accordingly, it can be concluded that a healthy professional environment and respect in relationships among the personnel can enhance professional self-concept and commitment to professional ethics.

Finally, it should be noted that enhancement of professional self-concept and propagation of ethical codes in every field can increase job satisfaction and feelings of being useful. Similarly, Fitzerglend and van Hooft (2000) believe that commitment to ethical codes is the equivalent of professional competence: if a nurse believes in the virtues of ethical codes, he/she is likely to do his/her best in any professional situation and try to improve the ethical standards in health care. Likewise, Carr (2008) believes that if we develop ethical virtues by military discipline, nurses will perform better in their profession, which is what the society expects from nursing. According to Mlinar, Tušak and Karpljuk's study (2009), too, nurses with higher scores of professional self-concept feel more responsible about their behavior and interactions with others. They show their satisfaction with their jobs in their environment and combine their best clinical performance with suitable ethical approaches.

Since the research context was limited to the teaching hospitals of Jahrom, Iran, the transferability of the results to other nurses and non-teaching hospitals is limited. Another is non-random sampling and cross-sectional data. The results need to be interpreted with caution as they may not be generalizable. Thus, it is suggested that complementary studies with larger samples be conducted. Future studies could add some qualitative data to compare contrast and enrich the findings.

## 5. Conclusion

The results of the study show that there is a significant relationship between nurses’ professional self-concept and commitment to professional ethics. In view of the rising interest in strategic approaches to professional ethics, it is recommended that those aspects of nursing which are related to commitment to professional ethics be taken into consideration. It appears that techniques that enhance nurses’ perception of themselves will increase their commitment to ethics, and vice versa. The more the authorities and nursing managers address these issues in their education and programs, the more successful they will be in training nurses who provide efficient and ethical care, which is the ultimate goal.
